# Identification of uterine ion transporters for mineralisation precursors of the avian eggshell

**DOI:** 10.1186/1472-6793-12-10

**Published:** 2012-09-04

**Authors:** Vincent Jonchère, Aurélien Brionne, Joël Gautron, Yves Nys

**Affiliations:** 1 INRA, UR83 Recherches Avicoles, F-37380, Nouzilly, France

**Keywords:** Ion, Mineral, Calcium, Transporter, Uterus, Eggshell, Chicken

## Abstract

**Background:**

In *Gallus gallus*, eggshell formation takes place daily in the hen uterus and requires large amounts of the ionic precursors for calcium carbonate (CaCO_3_). Both elements (Ca^2+^, HCO_3_^-^) are supplied by the blood via trans-epithelial transport. Our aims were to identify genes coding for ion transporters that are upregulated in the uterine portion of the oviduct during eggshell calcification, compared to other tissues and other physiological states, and incorporate these proteins into a general model for mineral transfer across the tubular gland cells during eggshell formation.

**Results:**

A total of 37 candidate ion transport genes were selected from our database of overexpressed uterine genes associated with eggshell calcification, and by analogy with mammalian transporters. Their uterine expression was compared by qRTPCR in the presence and absence of eggshell formation, and with relative expression levels in magnum (low Ca^2+^/HCO_3_^- ^movement) and duodenum (high rates of Ca^2+^/HCO_3_^-^ trans-epithelial transfer). We identified overexpression of eleven genes related to calcium movement: the TRPV6 Ca^2+^ channel (basolateral uptake of Ca^2+^), 28 kDa calbindin (intracellular Ca^2+^ buffering), the endoplasmic reticulum type 2 and 3 Ca^2+^ pumps (ER uptake), and the inositol trisphosphate receptors type 1, 2 and 3 (ER release). Ca^2+^ movement across the apical membrane likely involves membrane Ca^2+^ pumps and Ca^2+^/Na^+^ exchangers. Our data suggests that Na^+^ transport involved the SCNN1 channel and the Na^+^/Ca^2+^ exchangers SLC8A1, 3 for cell uptake, the Na^+^/K^+^ ATPase for cell output. K^+^ uptake resulted from the Na^+^/K^+^ ATPase, and its output from the K^+^ channels (KCNJ2, 15, 16 and KCNMA1).

We propose that the HCO_3_^-^ is mainly produced from CO_2_ by the carbonic anhydrase 2 (CA2) and that HCO_3_^-^ is secreted through the HCO_3_^-^/Cl^-^ exchanger SLC26A9. HCO_3_^-^ synthesis and precipitation with Ca^2+^ produce two H^+^. Protons are absorbed via the membrane’s Ca^2+^ pumps ATP2B1, 2 in the apical membrane and the vacuolar (H+)-atpases at the basolateral level. Our model incorporate Cl^-^ ions which are absorbed by the HCO_3_^-^/Cl^-^ exchanger SLC26A9 and by Cl^-^ channels (CLCN2, CFTR) and might be extruded by Cl^-^/H^+^ exchanger (CLCN5), but also by Na^+^ K^+^ 2 Cl^-^ and K^+^ Cl^-^ cotransporters.

**Conclusions:**

Our *Gallus gallus* uterine model proposes a large list of ion transfer proteins supplying Ca^2+^ and HCO_3_^- ^and maintaining cellular ionic homeostasis. This avian model should contribute towards understanding the mechanisms and regulation for ionic precursors of CaCO_3_, and provide insight in other species where epithelia transport large amount of calcium or bicarbonate.

## Background

**Figure 1:**
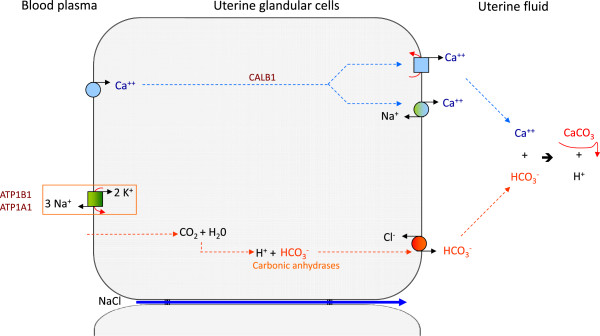
**Classic hypothesis concerning ion transfers in the hen uterus during eggshell calcification **[1,5,8-10]**.** Ca^2+^ entry in cell is passive via a Ca^2+^ channel, 28 kDa calbindin contributes to intracellular transfer and maintenance of a low Ca^2+^level. Ca^2+ ^secretion involving a Ca^2+ ^ATPase and a Ca^2+^/Na^+ ^exchanger. Carbonic anhydrase has a key role in providing carbonate from plasma CO_2_.

Biomineralisation is a process by which living organisms develop mineral structures to perform a variety of roles related to support, defence and feeding. Amongst these, a large number of animals (birds, molluscs, foraminifera, corals, sea urchins) mineralises by co-precipitation of calcium (Ca^2+^) and carbonates (CO_3_^2-^) to form a protective shell or a skeleton. The prerequisite for shell mineralisation is the supply of large amounts of Ca^2+^ and CO_3_^2-^ in a limited extracellular milieu by trans-cellular transport, requiring the presence of ion channels, ion pumps and ion exchangers. In *Gallus gallus*, eggshell formation takes place daily in the hen uterus and is one of the most rapid mineralisation processes [1]. It requires large amount of calcium carbonate (CaCO_3_) as the hen exports the equivalent of her body weight as eggshell in one year of egg production (>1.5 kg). Both elements (Ca^2+^ and HCO_3_^-^) are not stored in the uterus but are continuously supplied during eggshell formation by the blood plasma via trans-epithelial transport taking place across the uterine glandular cells [2-4]. Early studies determined the ion concentrations of the uterine fluid, which bathes the eggshell and changes during the sequential stages of calcification (Table1) [5], identified several proteins involved in ion transport [3,6,7], and recorded changes in ion fluxes across the uterine epithelium in response to ion transporter inhibitors [8-10]. These classic approaches led to a hypothesis concerning the mechanisms of ion transfer through the uterine glandular cells (Figure1; [1]). In hens, the Ca^2+^ blood (1.2 mM) and epithelial cell concentrations (10^-4^ mM), suggest that Ca^2+^ entry in cell is passive via a Ca^2+^ channel, which remains unidentified. The intracellular Ca^2+^ transport through the cell involves 28 kDa calbindin [3,11,12]. The 28 kDa calbindin expression is greatly upregulated during eggshell formation and falls after suppression of calcification (by premature egg expulsion), suggesting a very close relationship between uterine calbindin levels and Ca^2+ ^flux [11,13,14]. This protein could also take part in maintaining low intracellular Ca^2+^ to avoid cell death as observed in other species and tissues [15]. Ca^2+ ^secretion from epithelial cells to the uterine fluid is active involving a Ca^2+^ ATPase, the activity of which varies with the stage of eggshell calcification [4,7]. A recent study [16] identified and localized the plasma membrane Ca^2+ ^ATPase isoform 4 (PMCA4) in the apical membrane of epithelial cells of king quail. The disruption of sodium (Na^+^) re-absorption by specific inhibitors in perfused uterus or *in vitro* reduced Ca^2+^ secretion by 50% [9,17], revealing a strong relationship between Na^+^ and Ca^2+ ^transfers and therefore the putative presence of Na^+^/Ca^2+ ^exchangers in uterine cells. The Na^+^/K^+ ^ATPase responsible for Na^+ ^re-absorption in the plasma membrane is characterised and is upregulated during the period of shell calcification [18]. 

**Table 1:** **PH and ion concentrations in blood plasma, uterine fluid and epithelial cells during eggshell mineralisation **[5]

	**Blood plasma**	**Epithelial cells**	**Uterine fluid**	
			**8 h PO**	**18 h PO**
**Ions**	**[mM]**	**[mM]**	**[mM]**	**[mM]**
Ca^2+^	1.2	<0.0002	6	10
Na^+^	140	12	144	80
K^+^	4	139	12	60
HCO_3_^-^	23	12	60	110
pH	7.4	7.0-7.4	7.6	7.1
Cl^-^	130	4	71	45

The eggshell precursors are secreted in the uterine fluid where the eggshell mineralization daily takes place from 10 hours to 22 hours post ovulation (yolk entry in the oviduct). PO: post ovulation.

The second essential component of eggshell mineralisation is carbonate. Blood carbon dioxide (CO_2_) is provided in cells by passive diffusion through the plasma membrane [2,19]. In the uterine tubular gland cells, a family of key enzymes, the carbonic anhydrases (CA) [6] catalyses the hydration of CO_2 _to HCO_3_^- ^as confirmed by inhibition of HCO_3_^- ^production and secretion by acetazolamide, a CA inhibitor [9]. Chloride (Cl^-^) is absorbed by the uterus and any perturbation of Na^+^ flux by ouabain [9] reverses both the Na^+ ^and Cl^-^ fluxes, but reduces also HCO_3_^-^ secretion suggesting that its transfer is dependent on Cl^-^ via a Cl^-^/HCO_3_^- ^exchanger which has not been identified. Finally, the production of HCO_3_^- ^in tubular gland cells and of CO_3_^2- ^in the uterine fluid generates high levels of protons (H^+^) ions. The concomitant decrease in uterine and plasma pH during calcification reflects the reabsorption of H^+ ^[5].

Only a few genes and related proteins involved in uterine ion transfer have been identified to date. Our objective therefore was to use the recent information issuing from the chicken genome sequencing [20] and subsequent enrichment in the chicken gene/protein databases to identify uterine ion transport proteins. Use of a recent transcriptomic study revealing uterine genes related to eggshell calcification [21] and of the analogies with transporters previously described in mammalian tissues transferring large quantities of ions (intestine, kidney, pancreas) allows the identification of putative genes encoding proteins involved in uterine trans-epithelial ion transports. Confirmation of their presence in birds and evaluation of their involvement have been analysed by comparing gene expression in the uterus compared to the magnum (the oviduct segment responsible for the synthesis and secretion of egg white proteins) and the duodenum (Ca^2+ ^uptake and neutralization of stomach acid), where both Ca^2+ ^and HCO_3_^- ^trans-epithelial transfers are respectively low and high. The magnum and the uterus secrete a large amount of water, Na^*+*^ and Cl^- ^during the phase of hydration of egg albumen which takes place before the active phase of eggshell formation in the uterus [5,22]. By contrast, the duodenum is the proximal region of the intestine with a high capacity for Ca^2+ ^absorption [23] and secretes a large amount of HCO_3_^- ^for neutralization of gastric acidity [24,25]. An additional experimental approach was the comparison of gene expression in the uterus isolated from hens at the stage of eggshell formation, to those for which eggshell formation was suppressed by premature egg expulsion. We identified a large number of genes coding for ion transport and propose a general model describing the putative contribution and localisation of the ion transporters in the tubular gland cell of the hen’s uterus.

## Results

### Identification of uterine ion transporters

The first step of this work was to establish a list of ion transporters potentially involved in supplying eggshell minerals. The ion transfer model established in the *Gallus gallus* uterus (Figure 1) using physiological data [5,8,9] was used to produce a first list of genes encoding ionic transporter proteins. This approach was completed by using a recent transcriptomic study revealing genes overexpressed in the uterus (shell formation) compared to the magnum (egg white protein secretion) [21] and analogies with transporters previously described in mammalian tissues at the intestinal and kidney level [24,26]. A list of 37 genes was therefore selected as candidates possibly involved in uterine trans-epithelial ion transfers (Table2). To facilitate identification of candidates in the manuscript, we have only used the gene symbol for describing both genes and proteins. 

**Table 2:** Function of genes potentially involved in the ion transfer for supplying eggshell mineral precursors in hen uterus

**Name**	**Gene symbol**	**Functional data**	**Transfer type**
Transient receptor potential cation channel subfamily V member 6	TRPV6		Ca^2+ ^channel (plasma membrane)
Calbindin 28 K	CALB1	[11,14,28]	Ca^2+ ^intracellular transporter (intracellular)
Endoplasmic reticulum calcium ATPase 1	ATP2A1		Ca^2+ ^ATPases (endoplasmic & plasma membrane)
Endoplasmic reticulum calcium ATPase 2	ATP2A2		
Endoplasmic reticulum calcium ATPase 3)	ATP2A3		
IP3 receptor1	ITPR1		Ca^2+ ^channels (endoplasmic membrane)
IP3 receptor2	ITPR2		
IP3 receptor3	ITPR3		
Ryanodine receptor 1	RYR1		Ca^2+ ^channel (endoplasmic membrane)
Plasma membrane calcium-transporting ATPase 1 (PMCA1)	ATP2B1		Ca^2+^/H^+ ^exchanger (plasma membrane)
Plasma membrane calcium-transporting ATPase 2 (PMCA2)	ATP2B2		
Plasma membrane calcium-transporting ATPase 4 (PMCA4)	ATP2B4	[16]	
Sodium/calcium exchanger 1	SLC8A1		Na^+^/Ca^2+ ^exchanger (plasma membrane)
Sodium/calcium exchanger 3	SLC8A3		
Amiloride-sensitive sodium channel subunit alpha	SCNN1A	[31]	Na^+ ^channels (plasma membrane)
Amiloride-sensitive sodium channel subunit beta	SCNN1B	[31]	
Amiloride-sensitive sodium channel subunit gamma	SCNN1G	[31]	
Sodium/potassium-transporting ATPase subunit alpha-1	ATP1A1	[18]	Na^+^/K^+ ^exchanger (plasma membrane)
Sodium/potassium-transporting ATPase subunit beta-1	ATP1B1	[18]	
Solute carrier family 4 member 4	SLC4A4		Na^+^/HCO_3_^- ^co-transporters (plasma membrane)
Solute carrier family 4 member 5	SLC4A5		
Solute carrier family 4 member 7	SLC4A7		
Solute carrier family 4 member 10	SLC4A10		
Inward rectifier potassium channel 2	KCNJ2		Inward rectifiers K^+ ^channels (plasma membrane)
Inward rectifier potassium channel 5	KCNJ15		
Inward rectifier potassium channel 16	KCNJ16		
Calcium-activated potassium channel subunit alpha-1	KCNMA1		K^+ ^channel (plasma membrane)
Carbonic anhydrase 2	CA2	[6]	Catalyse HCO_3_^- ^formation (plasma membrane)
Carbonic anhydrase 4	CA4		
Carbonic anhydrase 7	CA7		
Solute carrier family 4 member 8	SLC4A8		HCO_3_^-^/Cl^- ^exchangers (plasma membrane)
Solute carrier family 4 member 9	SLC4A9		
Solute carrier family 26 member 9	SLC26A9		
Vacuolar H ATPase B subunit osteoclast isozyme	ATP6V1B2		H^+ ^pump (organelles and plasma membrane
Cystic fibrosis transmembrane conductance regulator	CFTR		Cl^- ^channel (plasma membrane)
Chloride channel protein 2	CLCN2		Cl^- ^channel (plasma membrane)
H(+)/Cl(−) exchange transporter 5	CLCN5		Cl^-^/H^+ ^exchanger (plasma membrane)

### Uterine expression of the 37 genes encoding ion transporters

The mRNA expression of 37 transporters was analysed by RT-PCR in the uterus, and three other ion secreting or absorbing epithelia (magnum, duodenum and kidney) and in muscle where no trans-epithelial ion transfer occurs (Additional file 1: Table 1). Amongst these 37 genes, mRNA expression was observed in the uterus for 34 genes. Three genes (the endoplasmic Ca^2+^ pump type 1(ATP2A1), two exchangers Na^+^ dependent (SLC4A8) or independent (SLC4A9) Cl^-^/HCO_3_^-^ were not expressed in the uterus and were not further studied.

A large majority of these 34 genes were also revealed in the duodenum. Conversely, SLC4A8 was expressed only in duodenum. Four genes were revealed only in the uterus and were not present in the magnum (TRPV6, CALB1, SCNN1B and SLC26A9) or in muscle (CALB1, SCNN1B, SLC4A10 and CLCN2). The 34 genes revealed in the uterus are candidates for supplying ions in the uterus.

### Comparative expression of ion transfer genes between uterus and other secreting tissues

**Figure 2:**
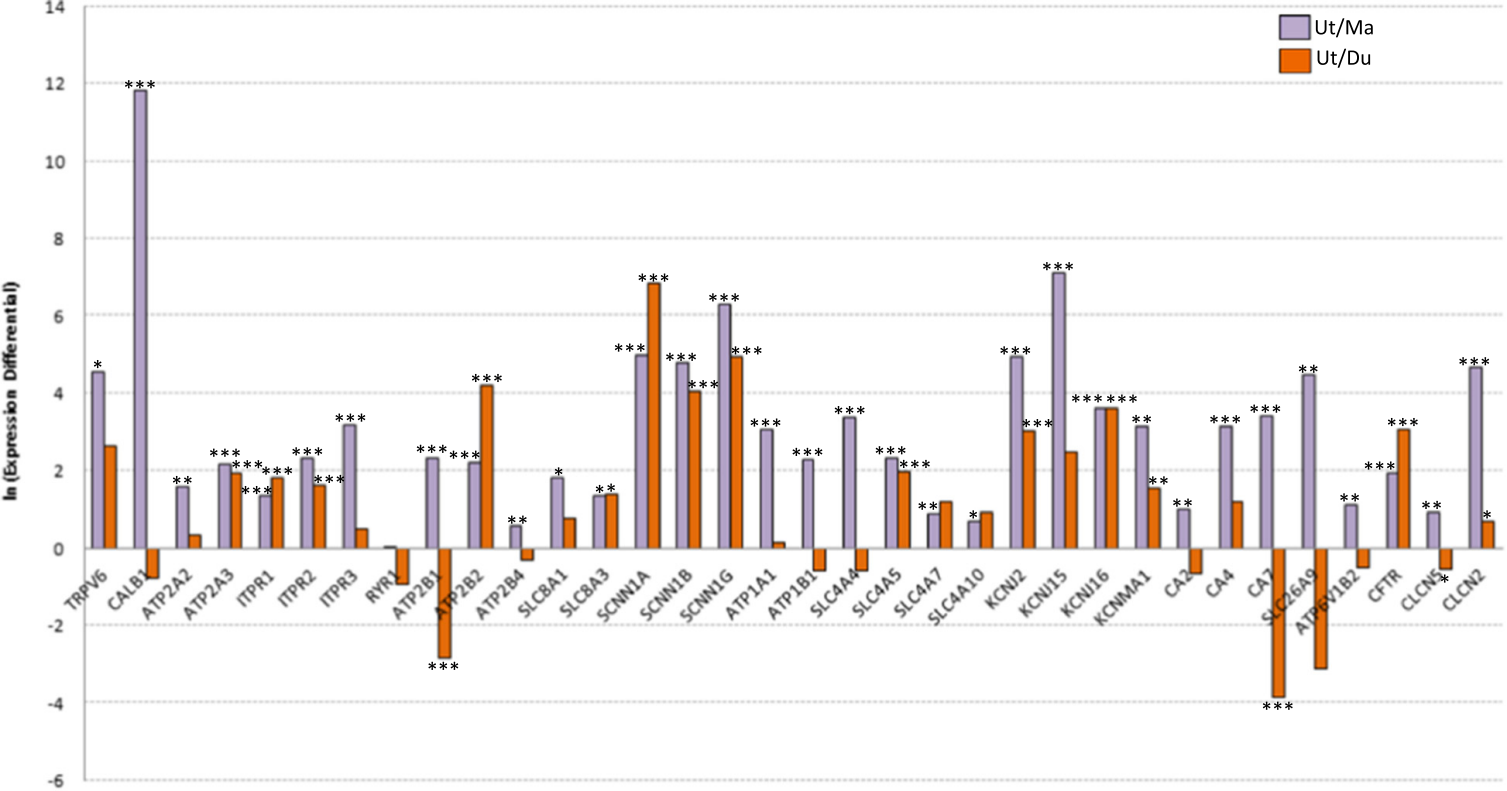
**Relative expression of genes coding ion transporters in chicken uterus compared to magnum or duodenum.** Gene expression of ion transporters for eggshell mineralisation were quantitatively evaluated by qRT-PCR in the uterus (eggshell formation) and compared to those of magnum and duodenum where Ca^2+ ^and HCO_3_^- ^trans-epithelial transport are at low and high levels, respectively.

The expression of the 34 genes encoding proteins potentially involved in uterine ion transfer were quantitatively evaluated by comparing their gene expression in the uterus to those of two other tissues (magnum, duodenum) where Ca^2+ ^and HCO_3_^- ^trans-epithelial transport are at low and high levels, respectively. After normalisation, the fold changes in gene expression between uterus *vs* magnum and uterus *vs* duodenum was statistically analysed (Figure2).

Amongst the 34 comparisons of gene expression between the uterus and the magnum, only one gene (the ryanodine receptor 1) was not differentially expressed. The 33 other genes showed higher levels of gene expression in the uterus than in the magnum (fold change of Ut/Ma up to 12 ln). Amongst these 33 genes, 16 genes (underlined in the following list) are not differentially expressed between uterus and duodenum suggesting they are equally important in both tissues able to absorb or secrete large amounts of Ca^2+ ^and HCO_3_^-^. These 33 gene candidates suspected to be involved in uterine ionic transfer corresponded to:

(1) Ca^2+^ transfer: TRPV Ca^2±^ channel (TRPV6), calbindin 28 kDa (CALB1), endoplasmic Ca^2±^ pump type 2 and 3 (ATP2A2, 3), inositol trisphosphate receptor type 1, 2, 3 (ITPR1, 2, 3), Ca^2±^ pumps PMCA type 1, 2 and 4 (ATP2B1, 2, 4) and Ca^2±^/Na^±^ exchanger type 1, 3 (SLC8A1, 3).

(2) Na^+^ transfer: amiloride-sensitive Na^**+**^ channel subunit α, β, and γ (SCNN1A, B, G), Na^±^/K^**±**^ transporting ATPase subunit α and β (ATP1A1, B1), Ca^2±^/Na^±^ exchanger type 1 and 3 (SLC8A1, 3), several Na^±^/HCO_3_^-^ co-transporters (SLC4A4, 5, 7, 10).

(3) K^+^ transfer: Na^±^/K^**±**^ transporting ATPase subunit α and β (ATP1A1, B1) and several K^±^ channels (KCNJ2, 15, 16, KCNMA1).

(4) HCO_3_^-^ production and transfer: CAs type 2, 4, 7, (CA2, 4, 7), an HCO_3_^-^/Cl^-^ exchanger (SLC26A9), and several Na^±^/HCO_3_^-^ co-transporters (SLC4A4, 5, 7, 10).

(5) H^+^ transfer: VH^±^ ATPase pump subunit B (ATP6V1B2), and Cl^-^/H^+^ exchanger (CLCN5).

(6) Cl^-^ transfer: CFTR channel (CFTR), Cl^-^ channel protein 2 (CLCN2), an HCO_3_^-^/Cl^-^ exchanger (SLC26A9) and a Cl^-^/H^+^ exchanger (CLCN5).

Fourteen genes amongst the 33 were overexpressed in the uterus compared with the duodenum. This overexpression of transporters in the uterus relative to the duodenum is indicative of genes whose function is more uterine specific. They corresponded to:

(1) Ca^2+^ transfer: endoplasmic Ca^2+^ pump type 3 (ATP2A3), inositol trisphosphate receptors (ITPR1, 2), Ca^2+^ pumps PMCA2 (ATP2B2) and Ca^2+^/Na^+^ exchanger type 3 (SLC8A3).

(2) Na^+^ transfer: amiloride-sensitive Na^**+**^ channel subunit α, β, and γ (SCNN1A, B, G), Ca^2+^/Na^+^ exchanger type 3 (SLC8A3), Na^+^/HCO_3_^-^ co-transporters (SLC4A5).

(3) K^+^ transfer: several K^+^ channels (KCNJ2, 16 and KCNMA1).

(4) HCO_3_^-^ production and transfer: Na^+^/HCO_3_^-^ co-transporters (SLC4A5).

(5) Cl^-^ transfer: Cl^-^ channel protein 2 (CLCN2) and CFTR channel (CFTR).

Three genes are underexpressed in the uterus compared with the duodenum suggesting that their function is more specific to the duodenum:

(1) Ca^2+^ transfer: Ca^2+^ pumps PMCA1 (ATP2B1).

(2) HCO_3_^-^ production and transfer: CA type 7 (CA7).

(3) H^+^ transfer and (4) Cl^-^ transfer: H^+^/Cl^-^ exchanger (CLCN5).

### Comparative expression of genes in the presence or absence of eggshell formation

**Figure 3:**
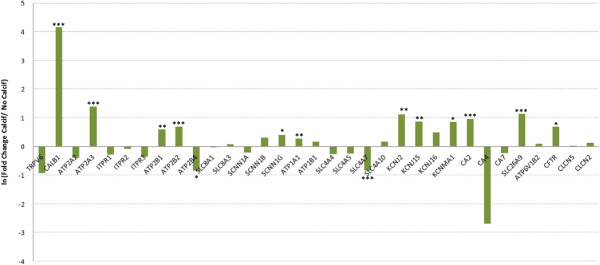
Comparison of ion transporter gene expression in the presence (Calcif) and absence of eggshell calcification (No Calcif).

This model was explored to reveal regulation of gene expression associated with the process of shell formation and to discern some of the ionic transport proteins more likely to be involved in supplying shell mineral precursors. We compared expression of these genes in the uterus when calcification takes place or after its suppression due to premature expulsion of the eggs for 3–4 consecutive days. The early egg expulsion eliminates the Ca^2+^ and HCO_3_^-^ requirement for shell formation, and eliminates the mechanical stimulation of the uterine wall due to the presence of the egg, which is known to upregulate expression of certain genes. Fold changes in gene expression between the calcifying or inactive uterus are presented in Figure3.

Twelve genes amongst 33 were overexpressed in the presence of eggshell calcification compared to hens in which shell formation had been suppressed (67 fold change):

(1) Ca^+2+^ transfer: 28 kDa calbindin (CALB1), endoplasmic Ca^2+^ pump type 3 (ATP2A3), and Ca^2+^ pumps PMCA2 (ATP2B1, 2).

(2) Na^+^ transfer**:** amiloride-sensitive Na^**+**^ channel subunit γ (SCNN1G) and Na^+^/K^+^ transporting ATPase subunit α (ATP1A1).

(3) K^+^ transfer: Na^+^/K^**+**^ transporting ATPase subunit α (ATP1A1) and the K^+^ channels (KCNJ2, KCNJ15 and KCNMA1).

(4) HCO_3_^-^ production and transfer: carbonic anhydrase CA type 2 (CA2), an HCO_3_^-^/Cl^-^ exchanger (SLC26A9).

(5) Cl^-^ transfer: the Cl^-^ channel (CFTR) and an HCO_3_^-^/Cl^-^ exchanger (SLC26A9).

In contrast, 2 genes corresponding to a Ca^2+^/H^+^ exchanger (ATP2B4) and to a Na^+^/HCO_3_^-^ co-transporter (SLC4A7) showed an underexpression when eggshell calcification takes place.

Nineteen of the 33 uterine genes did not show any fold change between these two physiological conditions:

(1) Ca^2+^ transfer: TRPV Ca^2+^ channel (TRPV6), endoplasmic Ca^2+^ pump type 2 (ATP2A2), inositol trisphosphate receptors (ITPR1, 2, 3), and Ca^2+^/Na^+^ exchanger type 1 and 3 (SLC8A1, 3).

(2) Na^+^ transfer: amiloride-sensitive Na^**+**^ channel subunit α and γ (SCNN1A, B), Na^+^/K^**+**^ transporting ATPase subunit β (ATP1B1), Ca^2+^/Na^+^ exchanger type 1 and 3 (SLC8A1, 3), Na^+^/HCO_3_^-^ co-transporters (SLC4A4, 5, 10).

(3) K^+^ transfer: Na^+^/K^**+**^ transporting ATPase subunit β (ATP1B1) and a K^+^ channel (KCNJ16).

(4) HCO_3_^-^ production and transfer: CA type 4, 7 (CA4, 7), several Na^+^/HCO_3_^-^ co-transporters (SLC4A4, 5, 10).

(5) Cl^-^ transfer: Cl^-^ channel protein 2 (CLCN2) and H^+^/Cl^-^ exchanger (CLCN5).

(6) H^+^ transfer: VH^+^ ATPase pump subunit B (ATP6V1B2) and H^+^/Cl^-^ exchanger (CLCN5).

## Discussion

Eggshell calcification in the avian uterus is one of the fastest mineralisation processes in the living world. The Ca^2+^ metabolism is intense in *Gallus gallus* hens which export a large amount of Ca^2+^ (2 g daily) and consequently there are numerous physiological adaptations to support this function [1,27-30]. In fact, an egg-producing hen shows a specific appetite for Ca^2+ ^a few hours before shell calcification is initiated and its capacity to absorb Ca^2+ ^in the intestine increases by 6-fold due to large stimulation of the active metabolite of vitamin D at the kidney level. The uterus acquires the capacity to transfer a great quantity of Ca^2+ ^and HCO_3_^- ^for supplying mineral precursors of the eggshell during less than 14 hours. This model is therefore particularly relevant to explore the mechanisms of mineral transport needed for the extracellular biomineralisation of the eggshell. In this study, we focused on intracellular ionic transporters and did not explore the proteins involved in their regulation. This process has been the object of many physiological and pharmacological works as reviewed by Nys [1] and Bar [30]. However, the molecular identification of ionic transporters remains incomplete in the uterus. Genome sequencing in human and other mammalian species has contributed to the molecular identification of genes and related proteins involved in ionic trans-epithelial transfer in the intestine and kidneys [24,26]. By using this literature and data provided by a recent high throughput analysis of chicken uterine genes related to eggshell calcification [21], we identified 37 putative genes encoding ion trans-epithelial transporters and tested their involvement in providing mineral precursors in the hen’s uterus. Analysis of their expression by RT-PCR, showed that 34 of these genes were expressed at the uterine level. In order to study their involvement in providing both Ca^2+ ^and HCO_3_^-^ for eggshell formation, the expression of these 34 genes in the uterus was quantified by qRT-PCR and compared with two other epithelia (magnum and duodenum) where Ca^2+ ^and HCO_3_^- ^transfers are respectively low and high. In addition, the expression of these genes was compared in the uterus during two situations: during eggshell calcification and when Ca^2+ ^and HCO_3_^- ^secretions were suppressed due to premature egg expulsion. These approaches allowed the identification of numerous transporting proteins providing minerals for shell formation in the hen’s uterus.

### Ca^2+ ^transfer

Ca^2+ ^is not stored in the uterus before eggshell calcification but comes from blood plasma by trans-epithelial transport. This Ca^2+ ^export is extremely rapid during calcification and corresponds to a consumption of the total plasmatic Ca^2+ ^pool every 12 min. Studies of Ca^2+ ^transfer *in vivo* using perfusion of uterus [8,9] and *in vitro* exploring the effects of inhibitors of ion ATPases or carbonic anhydrase [10,31], and ionic analysis of uterine fluid during eggshell formation [5], made it possible to build a first model of Ca^2+^ transfer in the uterus (Figure1): Ca^2+^, HCO_3_^- ^secretion and Na^+ ^reabsorption was considered to occur against their electrochemical gradient, to involve active intracellular transfer as shown by specific inhibitors [8-10] and to occur in the uterine glandular cells as revealed by immunohistochemistry of transport proteins [32]. Trans-epithelial transfer of Ca^2+^ occurs in three steps as observed in all transporting epithelia: Ca^2+^ influx through a downhill gradient, an intracellular Ca^2+ ^transport involving calbindin 28 kDa protein [33] and active output into the lumen through a Ca^2+ ^pump [4]. The high plasma Ca^2+ ^concentration (1.2 mM free Ca^2+^) relative to the uterine cell interior (10^-4^ mM free Ca^2+^) (Table1) suggests that the Ca^2+ ^entry into cells passively occurs via Ca^2+ ^selective channels present in the basolateral plasma membrane. In other tissues, such as intestine, kidney and plasma, TRPVs 5, 6 (Transient Receptor Potential Vanilloid) are epithelial channels that represent the principal pathway for Ca^2+^ uptake into the cell [26,34]. Our study showed that in *Gallus gallus*, only one gene [NCBI Gene ID: 418307; Swiss-Prot: TRPV6] is present. This channel is significantly overexpressed in the uterus compared with the magnum, where Ca^2+ ^transfer is low. Its uterine expression is similar to that of the duodenum where Ca^2+ ^absorption is also large. Cellular Ca^2+ ^influx might use a similar Ca^2+ ^channel, TRPV6, at the intestinal and uterine level but their localisation is hypothesized to differ according to the site of Ca^2+ ^influx, being located in the basal membrane in the uterus but in the apical membrane in the intestine. The uterine expression of TRPV6 is not however modified according to whether calcification takes place. The presence of other Ca^2+ ^channels cannot be ruled out as additional putative candidates. A recent transcriptomic study in our laboratory comparing uterine gene expression in hens with or without shell calcification revealed the presence of high expression of TRPC1, TRPP, TRPM7, TRPML1 and ORAI 1 (unpublished data, Brionne A, Nys Y and Gautron J).

An intracellular Ca^2+ ^buffer is crucial to keep the free cytosolic Ca^2+ ^concentration below toxic levels. Following Ca^2+ ^entry into the uterine glandular cell, several systems could contribute to intracellular transport of Ca^2+^, while maintaining the low but essential free Ca^2+ ^concentration for survival of the cell. In certain tissues, calbindin proteins, 9 kDa and 28 kDa in mammals [15] or 28 kDa in birds [3,35], are present at high cytosolic concentration and possess high Ca^2+^ binding capacity. Direct correlation has been demonstrated between their mucosal concentration and the efficiency of Ca^2+^ transfer in intestine and uterus under numerous experimental conditions [26,28,30]. It is generally accepted that calbindins facilitate the diffusion of intracellular Ca^2+ ^and serve as a Ca^2+ ^buffer needed for cell protection against Ca^2+ ^stress and accompanying apoptotic cellular degradation that is induced by a high intracellular Ca^2+ ^concentration [15,36,37]. In our study, we observed an elevated expression of calbindin 28 kDa in the uterus during calcification of an eggshell compared to the magnum (Figure2) and compared to the uterus with no shell in formation (fold difference in expression: 67) in agreement with previous studies [11,14,28]. This uterine calbindin 28 kDa is therefore associated with intracellular Ca^2+^ transport from the basal membrane of the glandular cells to the apical membrane where Ca^2+ ^is extruded into the uterine fluid.

An alternative system in mammals to maintain a low intracellular Ca^2+ ^concentration relies on the endoplasmic reticulum which contributes to Ca^2+^ homeostasis through its capacity for Ca^2+ ^uptake and storage [38,39]. The endoplasmic reticulum Ca^2+ ^ATPases (ATP2A1, 2, 3) play an active role in Ca^2+ ^uptake by this organelle (reaching 10 to 100 mM free Ca^2+^), while maintaining the cytoplasmic concentration at low concentrations of 10^-4^ mM free Ca^2+^. Amongst the three isoenzymes (Table2), only ATP2A2 and ATP2A3 were overexpressed in the uterus compared to the magnum. The absence of ATP2A1 expression fits with its predominant localisation in mammalian muscle in contrast to ATP2A2 and ATP2A3 which are expressed in numerous tissues [40]. The overexpression of ATP2A3 in the uterus compared to duodenum suggests a more crucial role of this transporter, the regulation of which remained to be explored.

The inositol 1, 4, 5-trisphosphate receptors (ITPR) are intracellular Ca^2+^ channels, localised mainly in the endoplasmic reticulum [41,42] and allowing the release of Ca^2+ ^from this organelle. The three isoforms (ITPR1, 2 3) were overexpressed in the uterus compared to the magnum but were not modified when comparing the presence or absence of calcification. The higher expression of ITPR1 and ITPR 2 in the uterus compared to the duodenum supports our hypothesis concerning their contribution to the regulation of intra-cellular Ca^2+^. The ryanodine receptors which are involved in muscle excitation-contraction coupling in mammalian tissues [38] are alternative channels for Ca^2+ ^release from the endoplasmic reticulum. RYR1 expression was revealed in the uterus, but there was no difference between the uterus, magnum or duodenum, suggesting a weak involvement in endoplasmic reticulum Ca^2+^ release. In conclusion, these observations of high expression of genes encoding ATP2A pumps and ITPR Ca^2+^ channels involved in Ca^2+ ^uptake and release in endoplasmic reticulum suggest the involvement of this organelle in intracellular Ca^2+ ^buffering in uterine glandular cells.

The last step of uterine Ca^2+^ trans-epithelial transport is output from the glandular cells, which occurs against a concentration gradient. Ca^2+ ^secretion towards the uterine fluid occurs via an active process, involving the Ca^2+^ ATPase [7,32,43]. This has recently been associated with the PMCA4 (plasma membrane ATPase Ca^2+^) [16]. Four isoenzymes (ATP2B1, B2, B3 and B4) of PMCAs pumps are identified in mammals [44]. Only three (ATP2B1, B2, B4) are conserved in birds. Each of these were overexpressed in the uterus compared to the magnum (Figure2). ATP2B2 was also overexpressed in the uterus compared to the duodenum, and in presence of the eggshell mineralisation (Figure3) suggesting a more active role in Ca^2+ ^secretion at the uterine level. In contrast, ATP2B1 and ATP2B4 were underexpressed in the uterus compared to duodenum and for ATP2B4 in presence of shell formation. In mammals, it is ATP2B1 which plays a more important role in intestinal Ca^2+ ^absorption [26,45]. In other bird species, Parker et al. [16] localized the plasma membrane Ca^2+^-transporting ATPase 4 (ATP2B4) in the apical membrane of uterine epithelial cells but did not explore the presence of ATP2B2 and its differential expression during calcification. In human osteoblasts, the isoforms 1 and 2 take part in the Ca^2+ ^supply necessary for bone mineralisation whereas the isoform 4 is not detected [46].

It was observed thirty years ago that the inhibition of Na^+^ transfer by Na^+^/K^+^ ATPase inhibitors considerably reduced Ca^2+^ secretion into the uterine lumen [9,17], showing a coupling between uterine Ca^2+^ secretion and Na^+ ^re-absorption. The uterine absorption of Na^+^ is revealed by the decreased Na^+ ^concentrations in the uterine fluid observed between the early stage of shell calcification and the end of calcification (Table1). These observations support the hypothesis that Na^+^/Ca^2+^ exchangers participate in the uterine Ca^2+^ secretion. The role of these transporters is clearly established at the mammalian intestinal and renal level [47]. Our study supported this mechanism for Ca^2+^ secretion in the chicken uterus, as both Na^+^/Ca^2+ ^exchangers (SLC8A1 and 3) were overexpressed in the uterus compared to the magnum, whereas their expression did not change in the presence or absence of eggshell mineralisation (Figures2 and 3). The mammalian exchangers allow the cell output of one Ca^2+^ ion against three Na^+^ ions at the basolateral membrane level. This transport is facilitated by the Na^+^ gradient, which provides the energy necessary for the Ca^2+ ^output against its gradient [34,47]. Similarly, the respective Na^+^ gradient between the cell (12 mM) and the uterine fluid (80 to 144 mM, Table1) may provide the bird uterus with the energy needed for the Ca^2+^ output towards the uterine fluid at the apical membrane of the glandular cells. Conversely, the unfavourable gradient of Na^+^ concentrations between blood (140 mM) and glandular cells at the basal membrane level will prevent Ca^2+^ uptake in the cells by exchange with Na^+^. Both Na^+^/Ca^2+^ exchangers (SLC8A1 and 3) are therefore predicted to be present only in the apical membrane of the uterine glandular cells. The co-expression of the SLC8A1 and 3 genes and of ATP2BX is observed in numerous Ca^2+^ transporting epithelia [48-51] but their respective involvements in Ca^2+^ flux has been questioned. Na^+^/Ca^2+^ exchangers have a weak affinity for Ca^2+^, but strong Ca^2+^ conductance. On the other hand, the Ca^2+^ ATP2BX pumps have a strong affinity for Ca^2+^, but a weaker conductance [26]. These data suggest that Ca^2+^ transport is mainly assured by the Na^+^/Ca^2+^ exchangers. In the hen uterus, the inhibition of the Na^+^/K^+^ ATPase led to a 60% decrease in Ca^2+^ transport *in vitro* or during uterine perfusion [9,17]. This observation underlines the importance of the Na^+^/Ca^2+ ^exchangers in the avian uterus.

**Figure 4:**
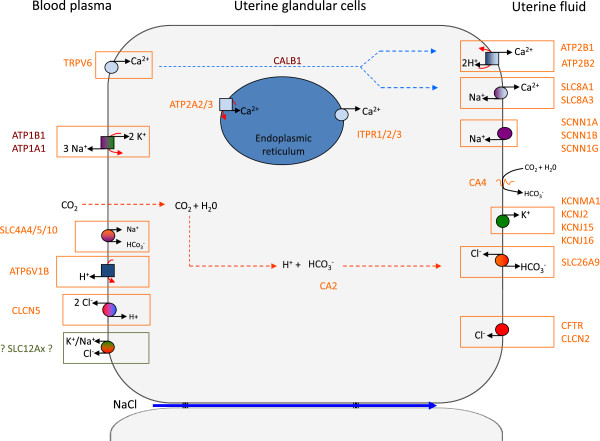
**New and general model describing uterine ion transporters during eggshell calcification.** The hypotheses concerning the transfer of ions through the uterine glandular cells are described in conclusion.

The information on uterine Ca^2+ ^transport is summarized in the model described in Figure4.

### Na^+^ transfer

During eggshell calcification, Na^+^ is absorbed from the uterine fluid into the blood plasma. This absorption resulting from the predominance of apical to basolateral flux relative to basolateral to apical flux, is partly due to the presence of the Na^+^/Ca^2+^ exchangers (SLC8A1 and 3), but a complementary system has been demonstrated by using epithelial Na^+^ channel blockers [31]*.* Amiloride-sensitive Na^+^ channels are essential in various epithelia [52]. Three subunits (SCNN1A, 1B, 1 G) of the Na^+ ^channel are overexpressed in the uterus compared to the magnum and to the duodenum (Figure2), suggesting the involvement of these transporters in Na^+^ uptake by the uterine glandular cells at the apical membrane. The γ subunit (SCNN1G) was overexpressed during shell calcification in contrast to the α and β subunits (SCNN1A, 1B) suggesting its predominant involvement in the uterus.

In the basolateral membrane, the Na^+^ glandular cell output towards plasma is active and occurs against a large electrochemical gradient (Table1). This is provided by the Na^+^/K^+^ ATPase, which is crucial in all animals for actively transporting Na^+^ out and K^+^ into the cell, and for maintaining the membrane potential and active transport of other solutes in intestine, kidney or placenta [34,53]. Its presence in the avian uterus and crucial role in ionic transfer during shell formation has been demonstrated [8-10,17]. *In situ* hybridization in the chicken uterus [18] showed that only the α1 subunit of Na^+^/K^+^ ATPase (ATP1A1), is present in the uterus whereas the α2 and α3 subunits (ATP1A2, A3) are absent. In this study, the α1 subunit (ATP1A1), but also the β1 subunit of Na^+^/K^+^ ATPase (ATP1B1), were overexpressed in the uterus compared to the magnum. We also confirmed the overexpression of α1 subunit of Na^+^/K^+ ^ATPase during the phase of calcification in contrast to the β1subunit of Na^+^/K^+ ^ATPase, in agreement with Lavelin et al. [18].

The possibility of an uptake of Na^+ ^from plasma into the uterine glandular cells at the basal membrane via Na^+^/HCO_3_^- ^co-transporters (SLC4A4, 5, 7, 10) is discussed in the section addressing HCO_3_^- ^transfer.

### K^+^ secretion

In the gastrointestinal or kidney epithelia, K^+^ channels provide the driving force for electrogenic transport processes across membranes and are involved in cell volume regulation or in secretory and reabsorptive processes. K^+^ channels are crucial for maintenance of body homeostasis and form the largest group of ion channels in mammals as more than one hundred thirty genes have been identified in human [54,55]. The chicken database revealed more than 80 such genes in birds. We explored only a limited number of K^+ ^channel candidates in chicken uterus by selecting 4 K^+^ channels overexpressed in uterus compared to their expression in magnum, as revealed in our hen transcriptomic study [21]. The increased K^+ ^concentrations in uterine fluid between early (8 hours) and late stages of calcification (Table1), demonstrates that uterine K^+^ net flow corresponds to a secretion into uterine fluid. A portion of K^+ ^secretion might be associated with the passive component of water secretion which occurs during the egg plumping at the early stage of shell calcification (up to 10 hours) but no experimental data has explored this contribution through a putative paracellular pathway. K^+^ uptake at the basolateral membrane, from the blood plasma towards the uterine glandular cells, should result from activities of the Na^+^/K^+ ^ATPases (ATP1A1, B1). By analogy with other intestinal, kidney, pancreatic, placenta, mammary glands or blood cells, we expect that K^+ ^channels present in the glandular cell will recycle K^+^ to allow functioning of the active Na^+^/K^+ ^ATPases. We tested 4 genes: three coding K^+ ^channels (KCNJ2, 15, 16) and one K^+ ^large conductance Ca^2+^ activated channel (KCNMA1), which could be involved in K^+^ cell output (Tables 1 and 3). The KCNJ2, 15, 16 channels belong to the KCNJ family (potassium inwardly-rectifying channel, subfamily J) which are stimulated by external K^+^ concentration. Their expressions are observed in numerous epithelia where K^+^ secretion occurs [54-56]. The KCNMs (K^+^ large conductance Ca^2+^ activated channels, subfamily M) participate in K^+ ^output in a large range of tissues and epithelia and are regulated by Ca^2+ ^cellular levels [54-56]. Our results showed that the expression of KCNJ2, 15, 16 and KCNMA1 K^+^ channels were higher in the uterus compared with the magnum. Moreover, the expression of KCNJ2, 15 and KCNMA1 (KCNM subunit α) was stimulated during calcification (Figure3). These results showed that KCNJ2, 15, 16, and KCNM K^+ ^channels are involved in the maintenance of potential membrane and K^+^ recycling during eggshell calcification and have therefore been introduced in the model (Figure4). We propose to localize them in the apical membrane but we have no evidence that they are absent from the basolateral membranes. 

**Table 3:** Primers used for RT-PCR and qRT-PCR of ion transporter genes

**Gene symbol**	**RefSeq accession**	**Forward primer**	**Reverse primer**
***TPRV6***	XM_416530	AACACCTGTGAAGGAGCTGGTGAG	TCTGCTGCTTGTTTTGTTGCC
***CALB1***	NM_205513	CAGGGTGTCAAAATGTGTGC	GCCAGTTCTGCTCGGTAAAG
***ATP2A1***	NM_205519	AAGGGGGGGTCTTTAAGGATGG	CAAACTGCTCCACCACCAACTC
***ATP2A2***	XM_415130	GCAGCTTGCATATCTTTTGTGCTG	CATTTCTTTCCTGCCACACTCC
***ATP2A3***	NM_204891	CAACCCCAAGGAGCCTCTTATC	GGTCCCTCAGCGTCATACAAGAAC
***ITPR1***	XM_414438	AATGGCAAAAGGCGAGGAAAGC	GGAGCAGCAGCAAGCGGG
***ITPR2***	XM_001235612	TGAGCATTGTGAGTGGCTTC	GTTGACCTGGCTGTCCAAAT
***ITPR3***	XM_418035	AGTACAACGTGGCCCTCATC	GTCGTGTCTGCTCTCCATGA
***RYR1***	X95266	GTTCCTCTGCATCATCGGCTAC	AATTGCTGGGGAAGGACTGTG
***ATP2B1***	XM_416133	CTGCACTGAAGAAAGCAGATGTTG	GCTGTCATATACGTTTCGTCCCC
***ATP2B2***	XM_001231767	TTACTGTACTTGTGGTTGCTGTCCC	GGTTGTTAGCGTCCCTGTTTTG
***ATP2B4***	XM_418055	GCTGGTGAAGTTGTCATCCGTC	TGCTCTGAAGAAAGCTGATGTTGG
***SLC8A1***	NM_001079473	GGATTGTGGAGGTTTGGGAAGG	CTGTTTGCCAGCTCGGTATTTC
***SLC8A3***	XM_001231413	GGAGAGACCACAACAACAACCATTC	AGCTACGAATCCATGCCCACAC
***SCNN1A***	NM_205145	GCTTGCCAGAAAACAGTCCCTC	AGTCAGACTCATCCAGGTCTTTGG
***SCNN1B***	XM_425247	ATGGAAGTAGACCGCAGT	GTTGTATGGCAGCACAGT
***SCNN1G***	XM_414880	CAAAAGGCACTTCACCCGTTTC	GGACAATGATCTTGGCTCCTGTC
***ATP1A1***	NM_205521	GCACAAAGAAGAAAAAGGCGAAGG	GGGTGGAGGTGTAAGGGTATTTG
***ATP1B1***	NM_205520	TCTGGAACTCGGAGAAGAAGGAG	GACGGTGAGCAACATCACTTGG
***SLC4A4***	XM_420603	GGAAAGCACCATTCTTCGCC	CCTCCAAAAGTGATAGCATTGGTC
***SLC4A5***	XM_423797	TGAACGTCTCCGCTACATCCTG	ACTTTATCCACCTGGCTGACTCC
***SLC4A7***	XM_418757	AAATTGCCAAGTTCGTGGTGG	GCGAAGCAAATGAGAAGTTACGG
***SLC4A9***	XM_001232427	TCCTGACTGGAGTCTCTGTCTTCC	AGGTGATCTGGCTGGTGTTTTG
***SLC4A10***	XR_026836	CGCTGATGACAGATGAGGTGTTC	GGTGGTTCTATTCGGATTGTTGG
***KCNJ2***	NM_205370	CCATTGCTGTTTTCATGGTG	TCCTGGACTTGAGGAGCTGT
***KCNJ15***	XM_425554	TGAGGGAAGGGAGACTCTGA	GCTTCCATCCTCACTGCTTC
***KCNJ16***	XM_425383	CATTCCTGTTCTCCCTGGAA	CATTTTAGCCAAGGCTGCTC
***KCNMA1***	NM_204224	GGGATGATGCGATCTGTCTT	GACAAACCCACAAAGGCACT
***CA2***	NM_205317	ATCGTCAACAACGGGCACTCCTTC	TGCACCAACCTGTAGACTCCATCC
***CA4***	XM_415893	GCTAACACATTTTTCCCCCTTCC	CTTTATAGCACATCGCATCAGCC
***CA7***	XM_414152	GCACAAGTCTTATCCCATTGCC	GCCGTTGTTGGAGATGTTGAGAG
***SLC4A8***	XM_001235579	AGAAGAAGAAGTTGGACGATGCC	GGTCAGTTCTGTCCTTGCTGTTCTG
***SLC26A9***	XM_425821	GCCTCTTCGATGAGGAGTTTGAG	CTGACCCCACCAAGAACATCAG
***ATP6V1B2***	XM_424534	ATTCTCTGCTGCTGGTTTGCCC	CATGGACCCATTTTCCTCAAAGTC
***CFTR***	NM_001105666	AAGAGGGCAGGGAAGATCAACGAG	CGGGTTAGCTTCAGTTCAGTTTCAC
***CLCN2***	XM_423073	CCTGGACACCAATGTGATGCTG	CACGAAGGTCTTCAGGGTGAGATAC
***CLCN5***	XM_420265	CGATTGGAGGAGTGCTCTTTAGTC	CAAAAGGATTGATGGAACGCAG

### HCO_3_^-^ production and transfer

Eggshell mineralisation results from the co-precipitation of Ca^2+^ and HCO_3_^-^. The bicarbonate precursor of the eggshell calcite is mainly derived from the blood carbon dioxide (CO_2_) which penetrates the uterine glandular cells by simple diffusion through the plasma membrane [2,19]. Carbonic anhydrases (CAs) [6] catalyse the hydration of intracellular CO_2_ to HCO_3_^-^, which is secreted into the uterine fluid. In the mammalian gastrointestinal tract, including pancreas, the cellular and membrane bound CAs are key enzymes allowing secretion or reabsorption of large amount of acid across the mucosa or protect epithelial cells from acid injury by secreting bicarbonate [24,25,57]. In all mammalian species, the duodenum buffers gastric acid secretion by producing intracellular HCO_3_^- ^from CO_2 _at a higher rate than the stomach or distal small intestine. The CO_2 _originates from intestinal lumen at the duodenal level but is provided from blood plasma via the respiratory system to the uterine tissue [1]. This study showed a larger expression of the cytosolic CA2 and 7 and of the membrane bound CA4 in the chicken uterus than in magnum (Figure2). No difference in expression was observed between the uterus and the duodenum for CA2 and 4. Cytoplasmic CA2 is the predominant CA in the duodenum, playing a major function in the hydration of CO_2_ to produce HCO_3_^- ^[25]. Similarly, we propose that CA2 plays a major role in the uterus to provide the carbonate precursor for the eggshell. CA7 is significantly underexpressed in the uterus compared to the duodenum, suggesting a secondary rule in HCO_3_^- ^uterine production. A major role for CA2 is supported by the overexpression of this CA gene in the presence of eggshell mineralisation, in contrast to CA4 and 7 (Figure3).

The HCO_3_^-^ produced by CA2 in uterine glandular cells must be then secreted into the uterine fluid to build the eggshell. In mammalian pancreas [57] and in duodenum [25] which secrete large amounts of HCO_3_^-^ towards the lumen, anion HCO_3_^-^/Cl^-^ exchangers (SLC4AX) have been located in apical membrane and Na^+^/HCO_3_^-^ co-transporters (SLC26AX) at the basolateral membrane [58,59]. In the bird uterus, there is a strong association between HCO_3_^-^ secretion and Cl^-^ transport [9,31] which supports the involvement of HCO_3_^-^/Cl^-^ exchangers. The HCO_3_^-^ flow through the uterine apical membrane is an electrogenic process which is facilitated by output of intracellular Cl^-^, via an exchanger of the SLC26 electrogenic family [31,57]. Our study confirmed the expression of a HCO_3_^-^/Cl^-^ exchanger (SLC26A9) in the uterus as shown in other epithelial cells [59]. SLC26A9 is suspected to have a role in intestinal HCO_3_^-^ secretion, in particular to neutralise gastric acidity [60,61]. Our results showed an overexpression of SLC26A9 exchanger in the uterus compared to the magnum or when the calcification takes place, whereas no variation of expression was observed between the duodenum and the uterus (Figure2). These observations suggest a common mechanism between both tissues and support the hypothesis of the involvement of this transporter in the supply of HCO_3_^-^ for eggshell calcification. Na^+^/HCO_3_^-^ co-transporter genes (SLC4A4, 5, 7, 10; [58,62]) are also expressed in the uterus and likely contribute to HCO_3_^-^transport. SLC4A4, 5, 7 and 10 showed higher expression in the uterus than in the magnum. An overexpression relative to the duodenum is observed only for SLC4A5, the three others being similarly expressed (Figure2). SLC4A7 is underexpressed in the uterus during calcification compared to its absence (Figure3) suggesting that involvement of this transporter is limited during the eggshell calcification process. In mammals, Na^+^/HCO_3_^-^ co-transporters mediate the electroneutral movement of Na^+^ and HCO_3_^-^ across the plasma membrane [58]. The ionic concentrations in the plasma and uterine glandular cells (Table1) show a favourable concentration gradient for uptake of these ions, supporting the localisation of these transporters in the basolateral membrane of uterine glandular cells to allow HCO_3_^-^ entry. However, previous studies [2,19] showed that the majority of HCO_3_^-^ used for the eggshell came from blood CO_2_ and only for a minor part from plasma HCO_3_^-^. Na^+^/HCO_3_^-^ co-transporters (SLC4A4, 5, 10) are likely to have a minor role in HCO_3_^-^ supply to the uterine glandular cells. The cystic fibrosis transmembrane conductance regulator (CFTR) contributes to fluid secretion from epithelial cells of the lung, pancreas and intestine, as shown in pathological situations associated with impaired fluid production, Cl^-^ and HCO_3_^-^ secretion due to defective CFTR [63,64] or in pharmacological studies of reproductive epithelium [65]. Its contribution to HCO_3_^-^ secretion is unlikely because of the unfavourable gradient or it is possibly indirect through regulation of HCO_3_^-^ transporters [65]. Its role as a Cl^-^ channel is discussed in the following section on Cl^-^. Studies using specific inhibitors and measuring Cl^-^ and HCO_3_^-^ flows are needed to quantify the contribution of HCO_3_^-^/Cl^-^ exchangers in HCO_3_^-^ uterine secretion.

### H^+^ transfer

HCO_3_^-^ production in the glandular epithelial cells, its secretion into uterine fluid and the co-precipitation of CO_3_^2-^ with Ca^2+^ leads to a progressive acidification of the uterine fluid and of glandular cells [1,5]. In fact, two H^+^ are produced for each CaCO_3_ formed. This metabolic acidosis is partially compensated by hyperventilation by the hen and by an increased renal H^+^ excretion [22].

The plasma membrane Ca^2+^ -transporting ATPases (ATP2B1, 2) of the apical membrane actively extrude Ca^2+^, as previously mentioned. However several lines of evidence have established that these pumps contribute to H^+^ re-absorption coupled to Ca^2+^ secretion [66,67]. The present study highlights their crucial role in Ca^2+^ secretion by uterine glandular cells during eggshell formation and therefore in H^+^ re-absorption from the uterine fluid through the apical membrane. Alternatively, the Na^+^/H^+^ exchangers have been shown to contribute to H^+^ output in the pancreatic duct which also secretes large amount of HCO_3_^-^. In a recent transcriptomic study of the uterus (unpublished data, Brionne A, Nys Y and Gautron J), we detected expression of various Na^+^/H^+^ exchangers (SLC9A 1, 2, 6, 7, 8, 9), supporting this possibility.

In this study, RT-PCR shows that the V H^+^ ATPase pump (VAT) is expressed in the bird uterus during calcification. In mammals, this VAT complex is made up of at least 14 subunits and allows transfer of H^+^ by hydrolysis of ATP [68,69]. This VAT is present in many membranes of organelles and also frequently in the plasma membranes of renal cells or osteoclasts [70]. VAT is therefore a good candidate for transferring protons to plasma in the hen uterine glandular cell, especially as this VAT was revealed in other species producing CaCO_3_ biominerals and shown to export H^+^ during mineralisation [71,72]. This proton ATPase extrudes H^+^ across the basolateral membrane of pancreatic duct epithelium [57] which is secreting high amounts of HCO_3_^-^ using mechanisms quite similar to uterine glandular cells. Our study reveals overexpression of the VAT subunit B in the uterus, which transfers large amounts of H^+^ compared with the magnum, where limited amounts of H^+^ are transferred. The VAT is likely to participate in H^+^ export from cytoplasm of the uterine cells to the blood plasma across the plasma membrane. The role of CLCN5 in H^+^ transfer is discussed in the ensuing Cl^-^ section.

### Cl^-^ transfer

The Cl^-^ concentrations decrease from 71 to 45 mM in the uterine fluid (Table1) when comparing the initial and late stage of eggshell calcification in parallel with changes of larger magnitude in Na^+^ concentrations. The high concentration of these ions observed at the early stage of calcification might result from the large secretions of water, Na^+^ and Cl^-^ which occurs during the plumping period (hydration of the egg white proteins), 6 to 10 h after ovulation possibly through a paracellular pathway [1]. These water and saline secretions are completed at the initiation of the rapid phase of shell formation, when Na^+^ and Cl^-^ net fluxes are inversed. The net flux of Cl^-^ is inhibited by acetazolamide, demonstrating the relationship between Cl^-^ transport and HCO_3_^-^ secretion derived from CAs activity [8,9,31], and the involvement of HCO_3_^-^/Cl^-^ exchangers of the SLC4 or SLC26 family [57,62,73]. Amongst the SLC4Ax HCO_3_^-^/Cl^-^ exchangers, we observed no expression of SLC4A8 and there is no evidence of any expression of SLC4A1, 2 or 3 in avian uterine transcriptomic study [21]. The role of SLC26A9 exchanger was previously discussed in the HCO_3_^-^ section. This exchanger is predicted to be located in the apical membrane of the uterine glandular cells and to contribute to Cl^-^ cell uptake during eggshell calcification as suggested in hens subjected to acetazolamide inhibitors [31] or in other species [57,59].

The CLCN2 channel, a family member of the CLCN (Cl^-^ channel), is relatively ubiquitous in epithelial cells and other cellular types [74,75]. It is considered to participate in various functions such as cellular volume regulation [76,77], cardiac activity regulation [78,79] and Cl^-^ trans-epithelial transfer [80,81]. Our study revealed that the CLCN2 channel is overexpressed in the uterus compared to the magnum or the duodenum (Figure2). The uterine fluid (>45 mM) and intracellular (4 mM) Cl^-^ concentrations are favourable to a Cl^-^ passive entry in uterine glandular cells. In parallel, another Cl^-^ channel, the cystic fibrosis transmembrane conductance regulator (CFTR) could also contribute to Cl^-^ entry in the cell as observed in numerous tissues [74]. In the chicken uterus, the CFTR channel is expressed at a higher level than in the magnum and the duodenum (Figure2). It is also overexpressed in the uterus during eggshell calcification (Figure3). The CLCN2 and CFTR channel are therefore probably expressed in the apical membrane and might contribute to Cl^-^ entry in the cell.

On the other hand, Cl^-^ output could be carried out by CLCN5, another member of CLCN family. Renal proximal tubule cells highly express the V H^+^ ATPase for acidification of endosomes and electroneutrality is ensured by transfer of Cl^-^ by CLCN5 [74]. The CLCN5 H^+^/Cl^-^ exchanger [75] has been localised mainly in organelle membranes but also in the plasma membrane. Our study revealed an overexpression of CLCN5 H^+^/Cl^-^ in the uterus compared to the magnum, so this channel might contribute to Cl^-^ output through the basal membrane. An alternative would be that Cl^-^ output relies on cation-coupled cotransport as observed in fish or mammals. The SLC12 family consists of Na^+^-K^+^-2Cl^-^ cotransporters and of K^+^-Cl^-^ electroneutral cotransporters, and are expressed either in kidney where they contribute to salt reabsorption, or more ubiquitously being involved in cell volume regulation [82-84]. In the chicken uterus, one Na^+^-K^+^-2Cl^-^ cotransporter (SLC12A2) and 4 K^+^-Cl^-^ cotransporters (SLC12A4, 7, 8, 9) are putative candidate, as expression of these genes is revealed in the chicken uterus transcriptome (unpublished data, Brionne A, Nys Y and Gautron J). In addition, furosemide, a blocker of Na^+^-K^+^-2Cl^-^ cotransporters, has been shown to decrease egg shell thickness [85].

## Conclusions

Initial studies on ion transfer in the uterus using physiological and pharmacological approaches provided a preliminary model of ion transfer contributing to the uterine Ca^2+^ and HCO_3_^-^ necessary for shell mineralisation (Figure1) [1,5,8-10,17]. The current approaches using knowledge gleaned from the chicken genome sequence and uterine transcriptomic expression data [21] identified numerous genes encoding putative transporters supplying the mineral precursors of eggshell mineralisation. We have used this information to build a model describing the ion supply mechanisms in the uterus, following a logical sequence for ion transfers for secretion of large amounts of Ca^2+^ and HCO_3_^-^ to form the eggshell (Figure4). This work identified 31 genes and related proteins involved in this process. It is consistent with preliminary hypotheses. Our analysis also revealed that analogies exist in the mechanisms of HCO_3_^-^ secretion by pancreatic duct cells and by duodenum, and to a lower extent with intestinal epithelial cells for Ca^2+^ movement, even if the Ca^2+^ flux is reversed between both uterus and duodenum.

The main steps of ion transfer in the hen’s uterus can be summarised (as presented in Figure4):

(1) Ca^2+^ secretion through epithelial glandular cells involves TRPV6 Ca^2+^ channel in the basolateral membrane (cell uptake entry), 28 kDa calbindin (CALB1, intracellular transfer), endoplasmic Ca^2+^ pumps type 2, 3 (ATP2A2, 3, uptake by endoplasmic reticulum), and inositol trisphosphate receptors type 1, 2, 3 (ITPR1, 2, 3, output from the reticulum). Ca^2+^ is then extruded from the glandular cells by the membrane’s Ca^2+^ pumps (ATP2B1, 2) and Ca^2+^/Na^+^ exchangers (SLC8A1, 3). The endoplasmic Ca^2+^ pumps, inositol trisphosphate receptors, and 28 kDa calbindin contribute to maintain a low intracellular free Ca^2+^ concentration essential for cell survival.

(2) Na^+^ transport involves three Na^+^ channels (subunits SCNN1A, 1B, 1 G; uptake in the cell), Na^+^/Ca^2+^ exchangers SLC8A1 and 3 (uptake in the cell) and the Na^+^/K^+^ ATPase (ATP1A1, ATP1B1, output from the cell).

(3) K^+^ uptake entry into the cell results from the Na^+^/K^+^ ATPase; the K^+^ channels (KCNJ2, 15, 16 and KCNMA1) contribute to its output release at the apical membrane.

(4) HCO_3_^-^ is mainly produced from CO_2_ by CA2 and to a lesser extent by CA4, and is also provided at a low level from plasma by the Na^+^/HCO_3_^+^ co-transporters (SLC4A4, 5, 10). HCO_3_^-^ is exported from the cell through the HCO_3_^-^/Cl^-^ exchanger SLC26A9.

(5) HCO_3_^-^ synthesis in the cell and co-precipitation of HCO_3_^-^ with Ca^2+^ in the uterine fluid produces two H^+^ which are transferred to plasma via the membrane Ca^2+^ pumps ATP2B1, 2 in the apical membrane and the VAT pump at the basolateral level.

(6) Cl^-^ ions in the uterine fluid enter the cell by the HCO_3_^-^/Cl^-^ exchanger SLC26A9 and by Cl^-^ channels (CLCN2, CFTR uptake in the cells), and might be extruded by Cl^-^/H^+^ exchanger (CLCN5), but also by Na^+^-K^+^-2Cl^-^ and K^+^-Cl^-^ cotransporters (SLC12Ax).

This model proposes a large but not exhaustive list of ionic transfer proteins involved in the supply of Ca^2+^ and HCO_3_^-^ or in maintaining cellular homeostasis (volume, electroneutrality). The model qualitatively describes putative mechanisms and cellular localisation of the candidates. These hypotheses relying on expression of the genes and on analogies with other tissues that transfer large amount of ions, need to be confirmed using immunochemistry for their cell localisation or by specific inhibition, to establish their relative contribution and understand their interaction and regulation. This avian model where huge amounts of Ca^2+^ and HCO_3_^-^ are exported daily following a precise spatial and temporal sequence should contribute to understanding the mechanism and regulation of ionic precursors of CaCO_3_ and provide insight for other species secreting a CaCO_3_ biomineral such as coral, molluscs, foraminifera or sea urchins.

## Methods

### Animals handling and housing

The experiment was conducted at the Unité Expérimentale Pôle d'Expérimentation Avicole de Tours (UEPEAT - INRA, Tours, France) according to the legislation on research involving animal subjects set by the European Community Council Directive of November 24, 1986 (86/609/EEC) and under the supervision of an authorized scientist (Authorization # 7323, J Gautron). Forty week old laying hens (ISA brown strain) were caged individually and subjected to a light/dark cycle of 14 hour light and 10 hour darkness (14 L:10D). The hens were fed a layer of mash as recommended by the Institut National de la Recherche Agronomique (INRA). Each cage was equipped with a device for automatic recording of oviposition time.

### Collection of laying hens oviduct tissues

Tissue samples (magnum, uterus, duodenum, kidney and *gastrocnemius*) were harvested in 8 hens while the egg was in the uterus during the active phase of calcification (16–18 hour post-ovulation). Additionally, uterine tissues were collected from 8 birds injected with 50 μg of F2-α prostaglandin during 4 consecutive days to expel the egg before mineralisation had begun (6 to 8 hours post ovulation). All tissue samples were quickly frozen in liquid nitrogen and stored at −80°C until RNA extraction.

### Determination of *Gallus gallus* cDNA sequences involved in mineral supply and design of primers

The list of ion transporters was established using recent transcriptomic data and *Gallus gallus* databases when available. The transporters not yet identified in chicken were identified using human orthologs in Swiss-Prot/TrEMBL and RefSeq databases. The corresponding human sequences were aligned to *Gallus gallus* Refseq database using BlastN algorithm an e-value cut-off of 10^-20^. Primers (Table3), were designed from the *Gallus gallus* using Mac vector software (MacVector, Cambridge, U.K.). The quality of the primers was tested by virtual PCR for dimerization and specificity using Amplify 3X software [86].

### RNA isolation, reverse transcription and classical

Total RNA was extracted from frozen tissue samples using a commercial kit (RNeasy Mini kit, Qiagen; Courtaboeuf, France) and simultaneously treated with DNase (RNase-free DNase set, Qiagen; Courtaboeuf, France) according to the manufacturer’s procedure. RNA concentrations were measured at 260 nm using a NanodropND 1000 (Thermo Fischer, Wilmington, Delaware, USA). The integrity of RNA was evaluated on a 2% agarose gel and with an Agilent 2100 Bioanalyser (Agilent Technologies, Massy, France). Only RNA samples with a 28S/18S ratio > 1.3 were considered for RT-PCR and qRT-PCR experiments. Total RNA samples (5 μg) were subjected to reverse-transcription using RNase H-MMLV reverse transcriptase (Superscript II, Invitrogen, Cergy Pontoise, France) and random hexamers (Amersham, Orsay, France). PCR was performed using primers (Table3) for 30 cycles at 60°C. The specificity of the PCR reaction was assessed by sequencing of PCR products (Cogenics, Meylan, France), and alignment of the sequences using BLASTN algorithm against the *Gallus gallus* RefSeq nucleic data bank*.*

### Quantitative RT-PCR (qRT-PCR)

Alternatively, cDNA sequences were amplified in real time using the qPCR Master mix plus for SYBR® Green I assay (Eurogentec, Seraing, Belgium) with the ABI PRISM 7000 Sequence Detection System (Applied Biosystems, France). To account for variations in mRNA extraction and reverse transcription reaction between samples, mRNA levels were normalized either to ribosomal 18S rRNA levels for each sample in the first series of comparison (magnum, uterus, and duodenum) or to TBP (TATA box binding protein) for each samples in the second series of comparison (comparison of expression in the uterus with and without mineralisation). The expression levels of 18S rRNA were measured using TaqMan Universal PCR Master Mix and developed Taq-Man assay for human 18S rRNA (Applied Biosystems, Courtaboeuf, France) as previously validated [87]. The PCR conditions consisted of an uracil-N-glycosylase preincubation step at 50°C for 2 min, followed by a denaturation step at 95°C for 10 min, and 40 cycles of amplification (denaturation for 15 sec at 95°C, annealing and elongation for 1 min at 60°C). A melting curve was carried out from 60 to 95°C for each sample amplified with SYBR® Green. Each run included triplicates of no template controls, standards and samples. Standards correspond respectively to a pool of the magnum, uterus, and duodenum RT products for the first series of experiments and of the uterus with and without mineralisation for the second series of comparison. The threshold cycle (Ct), defined as the cycle at which fluorescence rises above a defined base line, was determined for each sample and cDNA control. A calibration curve was calculated using the Ct values of the cDNA control samples and relative amount of unknown samples were deduced from this curve. The ratio value was calculated for each sample as sample/18 S rRNA in the first comparison (magnum, uterus, and duodenum) or sample/TBP in the second comparison (uterus with and without calcification). The log of the ratio was used for statistical analysis using the 5^th^ version of StatView, software (SAS Institute Inc. Cary, NC). A one-way analysis of variance was performed to detect differences (P < 0.05; 8 replicates/treatment) in gene expression amongst different conditions.

## Competing interests

The authors declare that they have no competing interests.

## Authors' contributions

VJ, JG contributed to the strategy, the experimental design, and planning of the study. VJ carried out the experiments and analyses, interpreted data and wrote the first draft of the paper. JG is the supervisor of VJ (Ph.D. student). AB contributed to the interpretation of data and to the writing of the paper. YN conceived the research program focused on identification of egg proteins. He was involved in the strategy, the experimental design, data interpretation and was fully involved in the writing of the paper. All authors have read and approved the final manuscript.

## Supplementary Material

Additional file 1: Table 1 RT-PCR of the candidate genes potentially involved in ion transfer in four secreting tissues and in muscle. Click here for file
