# Supplementary material for: Identification of uterine ion transporters for mineralisation precursors of the avian eggshell
Source: BMC Physiol. 2012 Sep 4;12:10. doi: 10.1186/1472-6793-12-10 (PMC3582589; doi:10.1186/1472-6793-12-10)
Supplement: Additional file 1: Table 1 — RT-PCR of the candidate genes potentially involved in ion transfer in four secreting tissues and in muscle. [file 1472-6793-12-10-S1.docx]

| **Gene symbol** | **Tissues** | | | | |
| --- | --- | --- | --- | --- | --- |
|  | **Magnum** | **Uterus** | **Duodenum** | **Kidney** | **Muscle** |
| TRPV6 | **-** | **+** | **+** | **+** | **+** |
| CALB1 | **-** | **+** | **+** | **+** | **-** |
| ATP2A1 | **-** | **-** | **-** | **-** | **+** |
| ATP2A2 | **+** | **+** | **+** | **+** | **+** |
| ATP2A3 | **+** | **+** | **+** | **+** | **+** |
| ITPR1 | **+** | **+** | **+** | **+** | **+** |
| ITPR2 | **+** | **+** | **+** | **+** | **+** |
| ITPR3 | **+** | **+** | **+** | **+** | **+** |
| RYR1 | **+** | **+** | **+** | **+** | **+** |
| ATP2B1 | **+** | **+** | **+** | **+** | **+** |
| ATP2B2 | **+** | **+** | **+** | **+** | **+** |
| ATP2B4 | **+** | **+** | **+** | **+** | **+** |
| SLC8A1 | **+** | **+** | **+** | **+** | **+** |
| SLC8A3 | **+** | **+** | **+** | **+** | **+** |
| SCNN1A | **+** | **+** | **+** | **+** | **+** |
| SCNN1B | **-** | **+** | **+** | **+** | **-** |
| SCNN1G | **+** | **+** | **+** | **+** | **+** |
| ATP1A1 | **+** | **+** | **+** | **+** | **+** |
| ATP1B1 | **+** | **+** | **+** | **+** | **+** |
| SLC4A4 | **+** | **+** | **+** | **+** | **+** |
| SLC4A5 | **+** | **+** | **+** | **+** | **+** |
| SLC4A7 | **+** | **+** | **+** | **+** | **+** |
| SLC4A9 | **-** | **-** | **-** | **+** | **-** |
| SLC4A10 | **+** | **+** | **+** | **+** | **-** |
| KCNJ2 | **+** | **+** | **+** | **+** | **+** |
| KCNJ15 | **+** | **+** | **+** | **+** | **+** |
| KCNJ16 | **+** | **+** | **+** | **+** | **+** |
| KCNMA1 | **+** | **+** | **+** | **+** | **+** |
| CA2 | **+** | **+** | **+** | **+** | **+** |
| CA4 | **+** | **+** | **+** | **+** | **+** |
| CA7 | **+** | **+** | **+** | **+** | **+** |
| SLC4A8 | **-** | **-** | **+** | **+** | **+** |
| SLC26A9 | **-** | **+** | **+** | **-** | **+** |
| ATP6V1B2 | **+** | **+** | **+** | **+** | **+** |
| CFTR | **+** | **+** | **+** | **+** | **+** |
| CLCN2 | **+** | **+** | **+** | **+** | **-** |
| CLCN5 | **+** | **+** | **+** | **+** | **+** |
